# Development of the First Integrated Silver Nitrate-Containing In Vitro Regeneration Approach for the Endangered *Tulipa montana* var. *chrysantha*

**DOI:** 10.3390/plants15203239

**Published:** 2026-10-21

**Authors:** Mohammad Gholami, Masoud Azadbakht, Mahboubeh Davoudi Pahnekolayi

**Affiliations:** 1 Ornamental Plants Research Group, Department of Landscape Design, Sana Institute of Higher Education, Sari 48161-18761, Iran; 2 Department of Biology, Sana Institute of Higher Education, Sari 48161-18761, Iran; 3 Research Group Phytochemistry and Biochemistry of Natural Compounds, Institute of Chemical, Environmental and Biosciences Engineering, Technische Universität Wien, 1060 Vienna, Austria

**Keywords:** bulbous plant, conservation, extinction, germplasm, in vitro organogenesis, native ornamental species, *Tulipa montana*

## Abstract

*Tulipa montana* Lindl. var. *chrysantha* (Boiss.) Wendelbo is a native Iranian tulip occurring in the highlands of the Alborz Mountains. This study aimed to establish an efficient in vitro regeneration protocol by optimizing explant disinfection, bulblet regeneration, and rooting conditions. Basal and distal bulb-scale, leaf, petal, and flowering-stem explants were evaluated using different sodium hypochlorite (NaOCl) exposure times and silver nitrate (AgNO_3_) concentrations. AgNO_3_ concentrations of 0, 100, 200, and 300 mg L^−1^ were tested during establishment; 300 mg L^−1^ resulted in the lowest contamination and was subsequently retained during regeneration. Leaf, petal, and flowering-stem explants did not regenerate, whereas bulb-scale explants produced regenerated bulblets. Combinations of 6-benzylaminopurine (BAP) or thidiazuron (TDZ) with 1-naphthaleneacetic acid (NAA) were evaluated. The highest number of regenerated bulblets was obtained with 2 mg L^−1^ TDZ + 1 mg L^−1^ NAA, while the greatest shoot length occurred with 1 mg L^−1^ TDZ + 1 mg L^−1^ NAA. The highest root number was obtained on ½ MS + 1 mg L^−1^ NAA, whereas the longest roots developed on ½ MS + 0.5 mg L^−1^ NAA. Approximately 80% of plantlets survived after one month of acclimatization.

## 1. Introduction

Tulips (*Tulipa* L.) are economically important ornamental bulbous plants widely cultivated as cut flowers, potted plants, and garden ornamentals. Their high ornamental value and commercial importance have stimulated the development of numerous cultivars and propagation systems aimed at producing uniform and high-quality planting material [1,2]. The genus *Tulipa* belongs to the family Liliaceae and comprises bulbous geophytes distributed mainly across Eurasia and North Africa. In addition to cultivated tulips, the genus includes numerous wild species with considerable morphological, ecological, and genetic diversity, which represent valuable resources for ornamental breeding and biodiversity conservation [3,4,5]. Consequently, propagation and conservation of wild *Tulipa* species are relevant not only to biodiversity preservation but also to the maintenance of genetic resources that may have future horticultural value.

Among the wild tulips occurring in Iran, *Tulipa montana* Lindl. var. *chrysantha* (Boiss.) Wendelbo is a native bulbous taxon associated with mountainous habitats, including the Alborz region [6,7]. Its attractive flowers and adaptation to mountainous environments give it potential value as a native ornamental genetic resource, although it is not cultivated on the commercial scale of established horticultural tulip cultivars. Similar to other wild tulip species, its value therefore extends beyond immediate commercial production to the conservation of native genetic resources and their potential use in future horticultural and breeding programs. Natural populations may be affected by habitat disturbance, grazing pressure, environmental changes, and other anthropogenic factors [8,9]. In addition, conventional vegetative multiplication of bulbous plants is generally slow and produces a limited number of daughter bulbs. These characteristics can restrict the availability of planting material and increase the importance of complementary propagation methods for rare or geographically restricted plant resources.

Tulips can be propagated sexually through seeds and vegetatively through daughter bulbs. Seed propagation is particularly useful for breeding and genetic diversification, but seedlings generally require a relatively long period to reach flowering and exhibit genetic variation, whereas vegetative multiplication through daughter bulbs maintains the genotype but may occur slowly [1,2,10]. In vitro culture provides an alternative or complementary approach by allowing controlled multiplication from selected plant material and has consequently been investigated for the propagation and conservation of several *Tulipa* species and cultivars. However, regeneration responses are strongly influenced by species and genotype, explant source, culture conditions, and the type and concentration of plant growth regulators (PGRs) [3,5,11,12,13]. Therefore, a protocol optimized for one *Tulipa* genotype cannot necessarily be transferred directly to another species or even to another cultivar.

Previous studies illustrate the considerable diversity of regeneration responses within the genus. In the cultivar ‘Merry Widow’, floral-stem explants exhibited regenerative responses on modified Murashige and Skoog (MS) medium containing 6-benzylaminopurine (BAP) and 1-naphthaleneacetic acid (NAA) [11]. Podwyszyńska and Marasek [12] further demonstrated differences among tulip cultivars in their responses to cytokinin and NAA treatments, emphasizing the influence of genotype on in vitro multiplication. In *Tulipa tarda*, bulb-derived and callus-derived materials have also been used for adventitious bulb formation. In one study, callus explants cultured on MS medium containing 0.5 μM BAP and 3% sucrose produced up to 36.6 adventitious bulbs per gram of callus tissue under dark conditions, demonstrating the importance of medium composition and environmental conditions for bulb formation [13].

Studies of other wild and rare *Tulipa* species further demonstrate the need for species-specific optimization. Nabieva and Gerasimovich [14] investigated the threatened *T. kaufmanniana* using immature zygotic embryos as primary explants. Chilling treatments combined with BAP or thidiazuron (TDZ) promoted shoot and bulblet formation, and TDZ treatment resulted in adventitious bulb formation in 48% of chilled embryos under the conditions tested, compared with 39% with the corresponding BAP treatment. The study also demonstrated that temperature regime and PGR treatment were important determinants of embryo development and bulblet formation [14].

More recently, Tagimanova et al. [1] investigated in vitro propagation of the rare endemic species *T. auliekolica* and *T. turgaica*. The two species differed in their temperature requirements for seed germination and in the timing of microshoot formation from bulb-derived explants. Culture of fragmented bulb segments on MS medium containing TDZ and NAA induced microshoot development, primarily from the basal region of the explants, while subsequent culture conditions promoted microbulb formation. Under favorable conditions, the two species produced approximately 5–6 microbulbs per microshoot-derived explant, whereas mature bulb formation was further influenced by auxin treatment [1]. These findings, together with previous studies of cultivated and wild tulips [3,5,11,12,13], demonstrate that explant source, genotype, temperature, PGR composition, carbohydrate concentration, and culture conditions can substantially affect regeneration and bulblet formation.

The developmental pathway of in vitro regeneration may also differ among *Tulipa* species and experimental systems. For example, both direct and indirect routes of adventitious bulb formation have been documented in *T. tarda*, whereas other studies have reported regeneration from embryo- or bulb-derived tissues under conditions that differ in explant source and culture history [15]. Therefore, observations of bulblet formation alone do not necessarily establish a particular developmental pathway. In the present study, the regeneration pathway was not investigated histologically; consequently, the observed responses are described as in vitro regeneration or bulblet regeneration rather than as direct organogenesis.

In addition to regeneration conditions, successful establishment of aseptic cultures is a critical prerequisite for the propagation of plants collected from natural populations. Field-derived explants may carry surface-associated or endogenous microorganisms, making contamination control particularly important during culture initiation. The effectiveness of surface sterilization depends on factors such as explant type, disinfectant concentration, and exposure duration, and excessive sterilization can adversely affect tissue viability. Optimization of these parameters is therefore especially important when working with native or rare plants for which the amount of available starting material may be limited [10,13].

Silver nitrate (AgNO_3_) has been incorporated into various plant tissue–culture systems because silver ions can interact with ethylene-related physiological processes and can influence plant tissue responses [16,17,18,19]. However, its effects are highly dependent on species, genotype, concentration, medium composition, and culture conditions. In the present study, AgNO_3_ was investigated specifically during explant establishment to evaluate its effect on contamination. The concentration associated with the lowest contamination was subsequently incorporated into the regeneration media. Because AgNO_3_ was not independently varied during the regeneration experiments, its specific contribution to subsequent regeneration responses cannot be separated from the effects of the other medium components. Accordingly, the role of AgNO_3_ in the present study is considered primarily in relation to contamination control and aseptic establishment rather than as an independently demonstrated regeneration-promoting treatment.

Although in vitro propagation has been investigated in several *Tulipa* species, information concerning the establishment and regeneration of native Iranian *T. montana* var. *chrysantha* remains limited. In particular, information is needed on the establishment of aseptic cultures from different explant types, the effects of sterilization conditions and AgNO_3_ concentration on contamination, the responses of bulb-scale and aerial explants to different cytokinin–auxin combinations, and the rooting and acclimatization of regenerated material. The substantial variation reported among *Tulipa* species and cultivars indicates that results obtained from commercial genotypes cannot necessarily be extrapolated to native taxa. A species-specific approach is therefore required to identify suitable conditions for the propagation of *T. montana* var. *chrysantha*.

Therefore, the present study aimed to develop and optimize an integrated in vitro regeneration and propagation system for *T. montana* var. *chrysantha*. First, the effects of explant type and sodium hypochlorite (NaOCl) exposure duration on contamination during culture establishment were evaluated for bulb-scale and aerial explants. AgNO_3_ concentrations were subsequently evaluated for their effects on contamination, and the concentration producing the lowest contamination was selected for subsequent culture. Regeneration was then investigated using separate experiments with BAP or TDZ, each combined with NAA, to determine suitable conditions for bulblet formation and shoot development. Rooting responses were subsequently evaluated using different MS medium strengths and NAA concentrations, followed by transfer of regenerated plants to ex vitro conditions to assess acclimatization. By addressing culture establishment, regeneration, rooting, and acclimatization as sequential stages, this study provides species-specific information for the in vitro regeneration and conservation-oriented propagation of the native Iranian *T. montana* var. *chrysantha*.

## 2. Results

### 2.1. Effect of Explant Type and Sodium Hypochlorite Exposure Time on Contamination Rate During Surface Sterilization

#### 2.1.1. Bulb-Scale Explants (Exp 1)

The first experiment evaluated the effects of explant type and exposure time to 2.5% sodium hypochlorite on contamination of distal and basal bulb scales. Analysis of variance showed that explant type, sodium hypochlorite exposure time, and their interaction significantly affected the number of contaminated explants (Explant type: *F* (1, 18) = 57.14, *p* ≤ 0.01; Exposure time: *F* (2, 18) = 36.00, *p* ≤ 0.01; Explant type × Exposure time: *F* (2, 18) = 4.00, *p* ≤ 0.05). The mean number of contaminated explants was significantly higher in basal scales (5.00 ± 0.00) exposed to sodium hypochlorite for 5 min than in distal scales (3.00 ± 0.00), but their difference was not significant.

Sodium hypochlorite exposure time also significantly affected contamination. The lowest mean contamination was recorded after 15 min of exposure in the distal bulb scale (0.33 ± 0.57), whereas the highest value occurred after 5 min (Figure 1(A1)). Thus, extending exposure to 2.5% sodium hypochlorite from 5 to 15 min considerably reduced contamination. The greater contamination of basal scales could be due to their greater exposure to soil and soil-borne microorganisms. Therefore, these results indicated that basal bulb scales were more difficult to sterilize than distal scales, and that a 15 min exposure to 2.5% sodium hypochlorite provided the most effective contamination control among the treatments tested. In addition to the number of contaminated explants, the contamination rate (%) was also determined. Both experimental factors and their interaction significantly affected contamination rate (Explant type: *F* (1, 18) = 57.14, *p* ≤ 0.01; Exposure time: *F* (2, 18) = 36.00, *p* ≤ 0.01; Explant type × Exposure time: *F* (2, 18) = 4.00, *p* ≤ 0.05), with the most effective treatment observed in distal bulb scales subjected to 15 min of NaOCl sterilization (Figure 1(A2)).

#### 2.1.2. Leaf, Petal, and Flowering-Stem Explants (Exp 2)

In the second experiment, the effects of explant type and exposure time to 1.5% sodium hypochlorite were evaluated in leaf, petal, and flowering-stem explants. Both factors and their interaction significantly affected the number of contaminated explants and contamination rate (explant type: *F* (1, 18) = 129.33, *p* ≤ 0.01; exposure time: *F* (2, 18) = 121.33, *p* ≤ 0.01; explant type × exposure time: *F* (2, 18) = 3.33, *p* ≤ 0.05). Among the explant types, flowering-stem segments showed the highest mean contamination after 5 min exposure to NaOCl (5.00 ± 0.00; 100% ± 0.00), whereas petals exposed for 15 min had the lowest (0.00 ± 0.00) (Figure 1(B1,B2)). The reduction in contamination with increasing exposure time was therefore evident across the explant types tested.

### 2.2. Effect of Silver Nitrate Concentration on Contamination Rate of Different Explant Types

#### 2.2.1. Bulb-Scale Explants (Exp 3)

Due to the considerable contamination remaining after the initial sterilization treatments, silver nitrate was subsequently evaluated as an additional antimicrobial treatment. In the third experiment, silver nitrate concentration, explant type, and their interaction significantly affected contamination of bulb-scale explants (explant type: *F* (1, 24) = 9.143, *p* ≤ 0.01; silver nitrate concentration: *F* (3, 24) = 6.85, *p* ≤ 0.01; explant type × silver nitrate concentration: *F* (3, 24) = 3.04, *p* ≤ 0.05). Increasing the silver nitrate concentration from 100 to 300 mg L^−1^ progressively reduced contamination. The highest mean number of contaminated explants (1.66 ± 1.15) was recorded at the basal bulb scales without treatment with AgNO_3_ or with 100 mg L^−1^, whereas contamination was eliminated (0.00 ± 0.00) at 200 and 300 mg L^−1^ at both distal and basal bulb scales (Figure 1(C1,C2)). The 200 and 300 mg L^−1^ treatments effectively reduced contamination. These findings showed that incorporating silver nitrate into the culture medium substantially improved contamination control and increased the availability of healthy explants for subsequent multiplication.

#### 2.2.2. Leaf, Petal, and Flowering-Stem Explants (Exp 4)

In the fourth experiment, silver nitrate concentration significantly affected contamination number and contamination rate of leaf, petal, and flowering-stem explants (explant type: *F* (2, 36) = 9.80, *p* ≤ 0.01; silver nitrate concentration: *F* (3, 36) = 12.26, *p* ≤ 0.01; explant type × silver nitrate concentration: *F* (6, 36) = 4.46, *p* ≤ 0.01). The mean number of contaminated explants was highest (2.00 ± 0.00) in the control treatment, while no contamination was detected at 300 mg L^−1^. The 100 and 200 mg L^−1^ treatments did not differ significantly; however, the 300 mg L^−1^ treatment was the most effective in eliminating contamination (Figure 1(D1,D2)).

Based on the combined results of the four sterilization experiments, optimized sterilization protocols have been selected for bulb and aerial explants. For bulb scales, the selected treatment included 70% ethanol for 30 s, Captan for 15 min, 2.5% sodium hypochlorite for 15 min, followed by culture on MS medium containing 300 mg L^−1^ silver nitrate, 300 mg L^−1^ cefotaxime, and 300 mg L^−1^ Captan. For leaf, petal, and flowering-stem explants, the selected protocol included 70% ethanol for 30 s, Captan for 15 min, 1.5% sodium hypochlorite for 15 min, and culture on MS medium containing 300 mg L^−1^ silver nitrate together with 300 mg L^−1^ Captan and 300 mg L^−1^ cefotaxime.

### 2.3. Effect of PGR Type and Concentration on In Vitro Multiplication of T. montana var. chrysantha (Exp 5)

The fifth experiment evaluated the effects of cytokinin type and concentration combined with NAA on regeneration of *T. montana* explants (bulb scales (distal and basal), leaf, petal, and flowering stem). Regeneration occurred only in basal bulb scales. The analysis showed significant differences among the treatments for the number of newly formed bulblets (NB), shoot length (SL), and regeneration percentage (RP%) (NB: cytokinin type: *F* (1, 54) = 2.66, *p* = 0.11; cytokinin concentration: *F* (2, 54) = 104.00, *p* ≤ 0.01; NAA concentration: *F* (2, 54) = 72.00, *p* ≤ 0.01); cytokinin type × cytokinin concentration × NAA concentration: *F* (4, 54) = 2.66, *p* ≤ 0.05; SL: cytokinin type: *F* (1, 54) = 10.26, *p* ≤ 0.01; cytokinin concentration: *F* (2, 54) = 181.81, *p* ≤ 0.01; NAA concentration: *F* (2, 54) = 124.35, *p* ≤ 0.01); cytokinin type × cytokinin concentration × NAA concentration: *F* (4, 54) = 4.35, *p* ≤ 0.01; RP%: cytokinin type: *F* (1, 54) = 9.30, *p* ≤ 0.01; cytokinin concentration: *F* (2, 54) = 109.92, *p* ≤ 0.01; NAA concentration: *F* (2, 54) = 159.76, *p* ≤ 0.01); cytokinin type × cytokinin concentration × NAA concentration: *F* (4, 54) = 2.84, *p* ≤ 0.05). For bulblet production, the highest mean NB (3.66 ± 0.57) was obtained with 2 mg L^−1^ TDZ + 1 mg L^−1^ NAA, whereas no bulblet formation was recorded in the control treatment (0.00 ± 0.00) (Figure 2A). When TDZ, BAP, and NAA were applied individually did not differ significantly from the control. The SL observed in regenerated plantlets was also significantly affected by treatment. The highest SL (1.83 ± 0.25) was recorded with 1 mg L^−1^ TDZ + 1 mg L^−1^ NAA, while the control produced no shoots (Figure 2B). Moreover, the control and several treatments containing any type of cytokinin and auxin alone did not differ significantly in this trait. Regarding RP%, the highest percentage (100% ± 0.00) was recorded in the same explants with the highest new bulb formation (Figure 2C). Thus, the combination of TDZ and NAA had a pronounced positive effect on bulblet formation, with 2 mg L^−1^ TDZ + 1 mg L^−1^ NAA producing the highest number of bulblets. In contrast, 1 mg L^−1^ TDZ + 1 mg L^−1^ NAA resulted in the highest length of the aerial part. Comparison of the two cytokinin experiments showed that TDZ was more effective than BAP for bulblet production, since the highest bulblet number obtained with TDZ + NAA (3.66 ± 0.57) exceeded that obtained with BAP + NAA (2.33 ± 0.47). We therefore identified TDZ in combination with NAA as the more effective cytokinin treatment for regeneration of *T. montana* explants.

### 2.4. Effect of Culture Medium Strength and NAA Concentration on Rooting of T. montana var. chrysantha (Exp 6)

Regenerated bulblets of suitable size were transferred to the rooting stage to evaluate the effects of basal medium strength and NAA concentration. The two factors were MS versus ½ MS medium and 0, 0.5, and 1.0 mg L^−1^ NAA. Root number (RN) and root length (RL) were measured at the end of *Exp 6*. Culture medium strength, NAA concentration, and their interaction had a significant effect on the RN produced (culture medium type: *F* (1, 18) = 45.00, *p* ≤ 0.01; auxin concentration: *F* (2, 18) = 54.60, *p* ≤ 0.01; culture medium type × auxin concentration: *F* (2, 18) = 4.20, *p* ≤ 0.05). The highest mean of RN (5.00 ± 0.00) was obtained on ½ MS medium containing 1 mg L^−1^ NAA, whereas the lowest (0.33 ± 0.57) was recorded on full-strength MS medium without any PGRs (Figure 2D). Furthermore, culture medium strength, NAA concentration, and their interaction had a significant effect on the RL (culture medium type: *F* (1, 18) = 16.66, *p* ≤ 0.01; auxin concentration: *F* (2, 18) = 38.00, *p* ≤ 0.01; culture medium type × auxin concentration: *F* (2, 18) = 4.66, *p* ≤ 0.05). The longest roots (0.46 ± 0.05) were obtained in ½ MS medium with 0.5 mg L^−1^ NAA, whereas the shortest roots (0.03 ± 0.05) were recorded in the MS treatment without any PGRs (Figure 2E). As a result, ½ MS supplemented with NAA was more favorable for root formation than full-strength MS, with 1 mg L^−1^ NAA producing the greatest number of roots.

### 2.5. Acclimatization of Regenerated Plantlets

Following rooting, healthy rooted plantlets were transferred to soil conditions for acclimatization. Approximately 80% of the transferred plantlets survived the acclimatization stage. During the first week, the plantlets were maintained under high humidity using plastic coverings, and irrigation was gradually increased during acclimatization. The surviving plantlets successfully adapted to ex vitro conditions, demonstrating that the in vitro regeneration and rooting protocol could produce plants capable of subsequent transfer to soil. Figure 3 indicates the in vitro regeneration process from the collection of mother explants from nature to the successful acclimatization stage.

## 3. Discussion

### 3.1. Optimization of Explant Sterilization and Aseptic Establishment of T. montana var. chrysantha

Successful establishment of aseptic cultures is a critical first step in the in vitro propagation of bulbous plants, particularly when explants are collected from plants growing under natural conditions [2]. In the present study, contamination was significantly affected by explant type and sodium hypochlorite exposure time. Among bulb-scale explants, basal scales showed greater contamination than distal scales, whereas among aerial explants, flowering-stem explants showed the highest contamination and petals the lowest. This variation is likely related to differences in tissue position, environmental exposure, and the microbial load associated with individual organs. Similar explant-dependent differences in sterilization requirements have been reported in *Tulipa*, where sodium hypochlorite and ethanol are commonly used, but their concentrations and exposure times vary considerably among species and explant types [20,21,22,23].

For bulb-scale explants, increasing exposure to 2.5% sodium hypochlorite from 5 to 15 min reduced contamination, and 2.5% sodium hypochlorite for 15 min was selected for subsequent establishment. This response is consistent with previous work on field-grown tulip material. For example, Amirova et al. [23] reported that increasing sodium hypochlorite concentration and exposure time improved sterilization efficiency in tulip material, although the exact species should be interpreted according to their original study rather than inferred from the bibliographic record alone. In another study, *Tulipa julia* bulbs required relatively strong sodium hypochlorite treatment, with 4–5% NaOCl for 30–35 min providing effective contamination control without visible damage to bulb tissues [24]. Similarly, axillary buds of *T. gesneriana* have been disinfected using 70% ethanol followed by 2% sodium hypochlorite for 20 min before in vitro culture [3]. These studies, together with the present findings, demonstrate that the effective disinfectant concentration and exposure time depend strongly on species, explant type, physiological condition, and microbial load.

The aerial explants examined in the present study also differed in their response to sodium hypochlorite effects. Flowering-stem explants showed the highest contamination, whereas petals showed the lowest, and increasing exposure to 1.5% sodium hypochlorite from 5 to 15 min reduced contamination. However, necrosis or tissue injury was not recorded as an independent quantitative variable in the present experiments. Therefore, although higher sodium hypochlorite exposure resulted in lower contamination, the present data do not allow a direct quantitative assessment of whether the most effective treatments also caused tissue injury. This distinction is important because increasing disinfectant concentration or exposure time can improve microbial elimination but may also reduce tissue viability. The *T. julia* study provides an example of species-specific tolerance, because no visible damage to bulb tissues was reported after treatment with 4–5% NaOCl for 30–35 min [24]. Thus, sterilization should be optimized not simply to obtain the lowest contamination rate, but to obtain the greatest proportion of clean and viable explants capable of subsequent regeneration.

Contamination control in the present study should be interpreted in the context of the combined antimicrobial treatment. Although increasing AgNO_3_ concentration was associated with changes in contamination, all AgNO_3_ treatments also contained Captan (300 mg L^−1^) and cefotaxime (300 mg L^−1^), following NaOCl surface disinfection. Consequently, the observed reduction in contamination reflects the performance of the combined treatment system rather than an independently demonstrated antimicrobial effect of AgNO_3_. In the present study, increasing AgNO_3_ concentration was associated with lower contamination, with 300 mg L^−1^ resulting in the lowest contamination among the concentrations tested during explant establishment. However, because the other antimicrobial agents were maintained at constant concentrations, their individual contributions and potential interactions with AgNO_3_ could not be quantified. Future experiments using appropriate single-agent controls and combinations of antimicrobial treatments are needed to distinguish their respective effects and identify the most effective treatment while assessing potential phytotoxicity. Moreover, the role of AgNO_3_ in the present study was primarily related to aseptic establishment. Because AgNO_3_ concentration was not independently varied during the regeneration experiment, its specific contribution to regeneration cannot be determined. Studies in other bulbous plants have demonstrated that silver responses are strongly concentration- and species-dependent [25,26,27]. For example, Isah et al. reported concentration-dependent effects of AgNO_3_ on morphogenesis of *Gladiolus hybridus*, while recent work with silver-based treatments in *Lilium* also demonstrated dose-dependent responses [28,29]. These studies support the need for caution when extrapolating the physiological effects of AgNO_3_ across species.

The relatively high nominal AgNO_3_ concentration used here should also be considered in relation to the chemical composition of MS medium. The nominal concentration of AgNO_3_ added to the medium does not necessarily correspond to the concentration of free Ag^+^ available to the explants because chloride and other components of MS medium can influence silver speciation, solubility, and bioavailability [30]. Ag^+^ may interact with chloride ions and potentially form poorly soluble AgCl. Because silver speciation and free dissolved Ag^+^ were not determined in the present study, the physiological effects associated with 300 mg L^−1^ AgNO_3_ should therefore be interpreted in terms of the nominal AgNO_3_ concentration, rather than as a defined free-Ag^+^ concentration.

Overall, the results demonstrate that sterilization requirements in *T. montana* var. *chrysantha* were strongly dependent on explant origin. Bulb scales required 2.5% NaOCl for 15 min, whereas aerial explants were adequately established using 1.5% NaOCl for 15 min, with 300 mg L^−1^ AgNO_3_ subsequently used for contamination control. This difference is biologically plausible because bulb tissues develop in close association with soil and may carry a greater microbial load than aerial tissues. Similar variation among organs has been reported in *Tulipa* micropropagation studies and reviews [21,22,23,24].

Sterilization may also influence the subsequent regenerative capacity of explants. Excessive chemical exposure can damage cells or tissues even when contamination is effectively eliminated, thereby reducing the number of explants that remain physiologically competent for regeneration. Conversely, insufficient sterilization can result in microbial growth that rapidly compromises the culture. The present study did not independently quantify necrosis or post-disinfection viability; therefore, a direct relationship between disinfectant intensity and regeneration capacity cannot be established. Nevertheless, the successful regeneration obtained from basal bulb-scale explants indicates that the selected establishment procedure preserved sufficient regenerative competence in this tissue. Future studies should therefore evaluate contamination, tissue injury, and subsequent regeneration simultaneously to identify the optimal balance between aseptic establishment and explant viability.

### 3.2. Effect of Exogenous Cytokinins and NAA on In Vitro Regeneration of T. montana var. chrysantha

A clear difference was observed between the explant types during the regeneration phase. No regeneration was obtained from the aerial explants (leaf, petal, and flowering-stem explants), whereas basal bulb-scale explants produced regenerated bulblets. This result indicates that regenerative competence under the conditions tested was strongly dependent on explant type.

Successful regeneration from aerial explants has nevertheless been reported in other *Tulipa* species and cultivars. For example, Abdul-Karim and Hamad [3] obtained adventitious bud formation from actively growing floral-stem segments of *T. gesneriana*. Regeneration was strongly dependent on the position of the explant: median floral-stem segments produced buds from 88% of explants, whereas apical and basal segments produced only 4% and 3%, respectively. The regenerated buds could subsequently develop into bulblets following a cold treatment. Similarly, Podwyszyńska and Marasek-Ciolakowska [12] obtained regeneration from flower–stalk explants of six tulip cultivars, with TDZ increasing the percentage of explants producing leaf-like structures to 70–100% under cultivar-specific optimum conditions.

The successful use of aerial tissues in these studies may be related to several factors that differed from the present experiment, including genotype, developmental stage, explant position, physiological condition of the donor plants, chilling treatment, and culture-medium composition. In the *T. gesneriana* study, explants were obtained from actively growing floral stems, and the median region was markedly more responsive than the apical or basal regions [3]. In the study of Podwyszyńska and Marasek [12], flower–stalk segments were obtained from cooled and subsequently forced bulbs, and the response was strongly genotype-dependent. In contrast, the present study used aerial tissues collected from a native population of *T. montana* var. *chrysantha*. Thus, the absence of regeneration from aerial explants in the present study should not be interpreted as evidence that aerial tissues are inherently non-regenerative in *Tulipa*. Rather, it indicates that the conditions tested here were insufficient to induce regeneration in these tissues.

In the present study, regeneration was subsequently obtained from basal bulb-scale explants under both BAP- and TDZ-containing treatments, whereas the hormone-free control did not regenerate. The regeneration response depended on the combination of cytokinin concentration and NAA concentration, demonstrating that an appropriate cytokinin–auxin balance was required for bulblet initiation. Comparable variation has been reported among other Tulipa species. In *T. tarda*, Maślanka and Bach reported bulb formation of 56–92% from non-chilled explants under suitable BAP-containing conditions [13]. More recent work by Maślanka demonstrated that light spectrum, sucrose concentration, and BAP concentration substantially influenced adventitious-bulb formation in *T. tarda* [2]. These findings emphasize that regeneration percentage cannot be attributed solely to cytokinin type or concentration because environmental and nutritional conditions also influence morphogenesis.

Recent work on the Kazakh tulips *T. auliekolica* and *T. turgaica* also demonstrates high but species-dependent regeneration capacity. Tagimanova et al. [1] reported shoot-formation frequencies of 95.7 ± 2.1% for *T. auliekolica* and 91.3 ± 2.0% for *T. turgaica* from bulb-derived explants. These values were numerically higher than those observed under several treatments in the present *T. montana* experiment; however, direct comparison should be made cautiously because the experimental systems, media, explant preparation, and definitions of the regeneration endpoint differed.

The number of regenerated bulblets per explant in the present study was also strongly affected by cytokinin–NAA combinations. The highest numerical value was obtained with 2 mg L^−1^ TDZ + 1 mg L^−1^ NAA, while 2 mg L^−1^ BAP + 1 mg L^−1^ NAA also produced a high regeneration response. Because the main effect of cytokinin type on the number of regenerated bulblets was not significant in the current analysis, the numerical maximum under TDZ should not be interpreted as evidence that TDZ was generally superior to BAP. Instead, the response should be interpreted in relation to the specific cytokinin–NAA combinations.

Previous *T. tarda* studies demonstrate that considerably higher bulblet numbers can be obtained under optimized conditions. Maślanka and Bach [31] reported substantial adventitious-bulb formation from bulb-derived explants, while Maślanka later reported a maximum of approximately 12.1 bulbs per explant under optimized light, sucrose, and BAP conditions [2,13]. These values illustrate the strong influence of culture environment on bulblet production. For *T. auliekolica* and *T. turgaica*, Tagimanova et al. [1] reported mean shoot numbers of 2.8 ± 0.3 and 1.7 ± 0.3 shoots per explant, respectively, from bulb-derived material. Subsequent microbulb formation was also strongly affected by sucrose concentration, reaching 5.96 ± 0.4 microbulbs per explant in *T. auliekolica* and 4.96 ± 0.5 in *T. turgaica* at 90 g L^−1^ sucrose. These results demonstrate that bulblet production may be strongly influenced by the culture stage and carbohydrate concentration and should therefore not be compared directly with the cytokinin–NAA experiment of the present study.

In the present study, the treatment that favored bulblet formation was not necessarily the treatment that produced the longest shoots. Lower cytokinin concentrations combined with NAA favored shoot elongation, indicating that bulblet initiation and shoot elongation were partially separable responses. Similar species-specific differences have been reported in other tulips. In *T. auliekolica* and *T. turgaica*, Tagimanova et al. [1] found that medium composition strongly affected shoot elongation, with maximum shoot lengths of 11.8 ± 1.5 cm and 8.8 ± 1.9 cm, respectively, under their most effective medium conditions. These values were obtained from seed-derived material rather than the same bulb-scale system used here and therefore provide comparative rather than directly equivalent data.

The results of Podwyszyńska and Marasek [12] similarly demonstrated that cytokinin concentration and genotype strongly affected tulip shoot development. Their flower–stalk study showed substantial differences among cultivars in regeneration and subsequent shoot multiplication, further supporting the conclusion that *Tulipa* species and genotypes cannot be expected to respond uniformly to a single PGR regime.

Taken together, comparison with previous studies indicates that regeneration of *Tulipa* is strongly influenced by species or genotype, explant type, developmental stage, PGR composition, sucrose concentration, light, and temperature. The present study therefore provides a species-specific regeneration system for *T. montana* var. *chrysantha*, with basal bulb-scale explants being more responsive than the aerial explants tested here. AgNO_3_ should not be considered an independently demonstrated regeneration-promoting factor in the present study. The concentration of 300 mg L^−1^ was selected because it provided the lowest contamination during establishment and was then maintained across the regeneration treatments. Consequently, its independent contribution to regeneration cannot be separated from the effects of the tested cytokinin–NAA treatments. The developmental pathway of bulblet formation also remains unresolved. Although callus-like and nodular tissues were observed in NAA-containing treatments, no histological analyses were performed. Therefore, the regenerated structures are described as products of in vitro regeneration rather than confirmed direct organogenesis.

### 3.3. Effect of Culture Medium Strength and NAA Concentration on Rooting of T. montana var. chrysantha

Root development is an important stage in the transition of regenerated plants from in vitro culture to ex vitro conditions [32]. In the present study, medium strength and NAA concentration affected both root number per shoot and root length. The present Discussion dataset indicates that ½ MS supplemented with 0.5 mg L^−1^ NAA produced the greatest root number and longest roots, whereas hormone-free full-strength MS resulted in the poorest response. These results indicate that reduced mineral strength combined with a moderate auxin concentration can favor root development in regenerated *T. montana* plants. Rooting responses reported for other *Tulipa* species demonstrate substantial variation in auxin requirements. In *T. gesneriana*, Salybekova et al. [33] reported 100% rooting, with an average of 3.4 roots per bulb, on MS medium containing 0.1 mg L^−1^ NAA + 0.3 mg L^−1^ IBA. Abdul-Karim and Hamad [3] subsequently reported that 1.0 mg L^−1^ NAA was optimal for rooting *T. gesneriana*, producing up to five roots per plantlet. Podwyszyńska and Sochacki [34] described a large-scale micropropagation system in which tulip shoots are first induced to form bulbs and the resulting bulbs are subsequently rooted in vivo, rather than through an extensive in vitro rooting stage. These studies demonstrate that rooting percentage, root number, and root length provide complementary information for evaluating the effectiveness of a micropropagation protocol.

However, rooting percentage was not recorded in the present study. Consequently, the rooting results obtained here cannot be directly compared with studies reporting rooting percentage. The present experiment evaluated root number per shoot and root length, which describe the characteristics of roots produced by responding shoots but do not indicate the proportion of regenerated shoots that successfully rooted. Although rooting percentage was not assessed in the present study, future investigations should include this parameter alongside root number and root length to provide a more comprehensive evaluation of rooting performance.

The rooting strategy used here also differs from some established tulip micropropagation systems, in which regenerated shoots are induced to form bulbs and the resulting bulbs are subsequently rooted in vivo. Such approaches demonstrate that an extensive in vitro rooting stage is not always necessary for tulip propagation. In the present study, roots were induced directly from regenerated *T. montana* bulblets under in vitro conditions, providing a continuous aseptic regeneration and rooting procedure.

The successful acclimatization of approximately 80% of regenerated plantlets after one month demonstrates the practical potential of the established in vitro regeneration protocol for transferring *Tulipa montana* var. *chrysantha* to ex vitro conditions. This survival rate represents an important milestone in developing a functional regeneration and acclimatization system for this native Iranian tulip and provides a foundation for future conservation-oriented propagation efforts. Further longitudinal studies should investigate long-term plant survival, bulb development, flowering induction, and field performance to determine the persistence and reproductive potential of regenerated plants under ex vitro and natural conditions. These investigations will help establish the protocol’s broader applicability to the propagation and conservation of this species.

## 4. Materials and Methods

### 4.1. Plant Material and Collection Site

Plant material of the native Iranian tulip *Tulipa montana* var. *chrysantha* (Bioss.) Wendelbo was used as the experimental genotype. The species belongs to the section *Clusianae* Baker and subgenus *Leiostemones* Boiss. Bulbs and vegetative organs were collected from a natural population in the Alborz Mountains of Iran during the summer season. The collection site was located in the Asara region, approximately 50 km from Karaj and 26 km from Tehran, at 36°02′36″ N and 51°18′50″ E, with an elevation of approximately 2134 m above sea level. Morphological characteristics recorded for the collected plants included egg-shaped bulbs approximately 2–3 cm in diameter, linear leaves, and solitary flowers. The plants were described as growing naturally on dry mountain slopes at elevations of up to approximately 3200 m.

### 4.2. Explant Preparation and Surface Sterilization

Bulbs and vegetative tissues, including leaves, petals, and flowering stems, were used as explant sources. The outer bulb scales and basal plates were removed from the bulbs before explant preparation. For bulb-scale explants, the basal region was excised from the lower portion of the scale immediately above the basal plate; therefore, the basal plate itself was not included in the explant. The distal region was excised from the upper portion of the scale. The middle region was located between the basal and distal regions and was not used in the sterilization experiments. Before surface sterilization, the basal plate was removed from bulb scales and the bulbs were not cut; then the plant materials were washed under running tap water for 1 h. They were then subjected to the sterilization treatments described below. All experiments were conducted using three independent replicates. Unless otherwise stated, five explants were cultured per replicate, giving a total of 15 explants per treatment in Experiments 1–4.

#### 4.2.1. Effect of Sodium Hypochlorite Exposure Time on Bulb-Scale Explants (Exp 1)

The first experiment evaluated the effects of bulb-scale region and sodium hypochlorite (NaOCl) exposure time on explant contamination. Distal and basal bulb-scale explants were exposed to 70% ethanol for 30 s, followed by 2% Captan for 15 min and 2.5% NaOCl for 5, 10, or 15 min. The explants were subsequently rinsed three times with sterile distilled water and transferred to MS culture medium. Bulb-scale explants were cut to approximately 1.5 × 1.5 cm before culture. The experiment was arranged as a 2 × 3 factorial experiment in a completely randomized design (CRD), with bulb-scale region (basal and distal) and NaOCl exposure time (5, 10, and 15 min) as the two factors, resulting in six treatment combinations. Each treatment consisted of three independent replicates with five explants per replicate (15 explants per treatment). Contamination was evaluated after 7 days of culture. The number of contaminated explants was recorded for each treatment, and the contamination rate was calculated as:

Contamination rate (%)=NumberofcontaminatedexplantsineachvesselreplicateTotalnumberofcultivatedexplantsineachreplicatevesel×100

#### 4.2.2. Effect of Sodium Hypochlorite Exposure Time on Vegetative Explants (Exp 2)

The second experiment evaluated the effect of NaOCl exposure time on contamination of leaf, petal, and flowering-stem explants. The explants were washed under running tap water for 1 h, cut into approximately 2 × 2 cm segments, and treated with 70% ethanol for 30 s, followed by 1% Captan for 10 min and 1.5% NaOCl for 5, 10, or 15 min. The explants were then rinsed three times with sterile distilled water and transferred to MS culture medium. The experiment was arranged as a 3 × 3 factorial experiment in a CRD, with explant type (leaf, petal, and flowering stem) and NaOCl exposure time (5, 10, and 15 min) as the two factors, resulting in nine treatment combinations. Each treatment consisted of three independent replicates with five explants per replicate (15 explants per treatment). Contamination was evaluated after 7 days of culture. Contamination rate (%) was calculated using the formula described for Exp. 1.

#### 4.2.3. Effect of Silver Nitrate on Bulb Explant Contamination Rate (Exp 3)

Based on the results of Exp. 1, the selected sterilization procedure was used to evaluate the effect of silver nitrate (AgNO_3_) concentration on contamination of bulb-scale explants. Basal and distal bulb-scale explants were treated with 70% ethanol for 30 s, 2% Captan for 15 min, and 2.5% NaOCl for 15 min, followed by three rinses with sterile distilled water. The sterilized explants were cultured on MS medium containing 0, 100, 200, or 300 mg L^−1^ AgNO_3_, together with 300 mg L^−1^ Captan and 300 mg L^−1^ cefotaxime. A 500 mg L^−1^ AgNO_3_ stock solution was prepared, filter-sterilized, covered with aluminum foil, and stored under refrigerated conditions. After autoclaving and cooling of the culture medium, the appropriate volume of sterile AgNO_3_ stock solution was aseptically added to obtain the required final concentrations. Thus, AgNO_3_ was not autoclaved. Captan was incorporated into the culture medium before autoclaving, whereas cefotaxime was prepared as a stock solution, filter-sterilized, and aseptically added to the autoclaved and cooled medium. The experiment was arranged as a 2 × 4 factorial experiment in a CRD, with bulb-scale region (basal and distal) and AgNO_3_ concentration (0, 100, 200, and 300 mg L^−1^), resulting in eight treatment combinations. Each treatment consisted of three independent replicates with five explants per replicate (15 explants per treatment). Contamination was evaluated after 7 days of culture. The number of contaminated explants and contamination rate (%) were recorded for each treatment. The concentration resulting in the lowest contamination was subsequently selected for the regeneration experiments.

#### 4.2.4. Effect of Silver Nitrate on Leaf, Petal, and Flowering-Stem Explant Contamination (Exp 4)

Based on the results of Exp. 2, the selected sterilization procedure was used to investigate the effect of AgNO_3_ concentration on contamination of leaf, petal, and flowering-stem explants. Following the initial 1 h washing period, the explants were treated with 70% ethanol for 30 s, 1% Captan for 10 min, and 1.5% NaOCl for 15 min, followed by three rinses with sterile distilled water. Leaf, petal, and flowering-stem explants, approximately 2 × 2 cm, were cultured on MS medium containing 0, 100, 200, or 300 mg L^−1^ AgNO_3_, together with 300 mg L^−1^ Captan and 300 mg L^−1^ cefotaxime. AgNO_3_ was prepared and incorporated into the medium as described for Exp. 3. The experiment was arranged as a 3 × 4 factorial experiment in a CRD, with explant type (leaf, petal, and flowering stem) and AgNO_3_ concentration (0, 100, 200, and 300 mg L^−1^), resulting in 12 treatment combinations. Each treatment consisted of three independent replicates with five explants per replicate (15 explants per treatment). Contamination was evaluated after 7 days of culture, and contamination rate (%) was calculated as described above.

In Experiments 3 and 4, AgNO_3_ was evaluated at concentrations of 0, 100, 200, and 300 mg L^−1^ in culture media containing Captan (300 mg L^−1^) and cefotaxime (300 mg L^−1^). These agents were included at constant concentrations across the AgNO_3_ treatments. Accordingly, the experiments evaluated the effect of varying AgNO_3_ concentration within a combined antimicrobial system that also involved the preceding NaOCl surface-disinfection treatment. Because Captan and cefotaxime were not independently varied, the individual contributions of these agents and their potential interactions with AgNO_3_ could not be determined. The observed contamination levels were therefore interpreted in the context of the combined antimicrobial treatment rather than as an isolated effect of AgNO_3_.

### 4.3. In Vitro Regeneration and Bulblet Production

Following the sterilization experiments, healthy aseptic explants were transferred to the regeneration stage. The explants were maintained under the establishment conditions for 4 weeks before the cytokinin experiments were initiated. The AgNO_3_ concentration that produced the lowest contamination during explant establishment was used in all subsequent regeneration media. Thus, AgNO_3_ was not independently tested as a factor in the regeneration experiments.

#### 4.3.1. Effect of Cytokinin Type and Concentration (Exp 5)

Following establishment of aseptic cultures, several explants (distal and basal bulb explants, leaf, petal, and flowering stem) were transferred to the regeneration/multiplication stage. The effects of two cytokinins, 6-benzylaminopurine (BAP) and thidiazuron (TDZ), each in combination with the auxin 1-naphthaleneacetic acid (NAA), were evaluated. BAP and TDZ were each tested at concentrations of 0, 1, and 2 mg L^−1^ in combination with NAA at 0, 1, and 2 mg L^−1^. Thus, the experiment comprised 18 treatment combinations corresponding to two cytokinin types × three cytokinin concentrations × three NAA concentrations. The experiment was arranged in a CRD with three independent replicates and three explants per replicate (nine explants per treatment). Based on the results of Exp 4, in which 300 mg L^−1^ AgNO_3_ resulted in the lowest contamination during explant establishment, this concentration was selected for the subsequent regeneration experiment. Therefore, all regeneration media contained 300 mg L^−1^ AgNO_3_ in addition to the respective cytokinin and NAA treatments. The regeneration responses were evaluated based on the number of regenerated bulblets per explant (NB), shoot length (SL), and regeneration percentage (RP%). The number of regenerated bulblets per explant was expressed in relation to the highest number of newly formed bulblets produced by an explant under each replication. Regeneration percentage was calculated as:

$$\mathrm{Regeneration Percentage}\;(\mathrm{RP}\%)=\frac{\mathrm{Explants that regenerated bulbs}\;(B)}{\mathrm{Total Explants cultured}\;(N)}\times 100$$

#### 4.3.2. Rooting of Regenerated Bulblets (Exp 6)

Bulblets of suitable size obtained during the regeneration stage were transferred to the rooting phase. The effects of basal medium strength and NAA concentration on root formation were evaluated using full-strength MS and half-strength MS (½ MS) media supplemented with 0, 0.5, or 1.0 mg L^−1^ NAA. The rooting experiment was arranged as a two-factor factorial experiment in a completely randomized design (CRD), with basal medium strength (two levels: full-strength MS and ½ MS) and NAA concentration (three levels: 0, 0.5, and 1.0 mg L^−1^), resulting in six treatment combinations. Each treatment consisted of three independent replicates, with three regenerated bulblets per replicate (nine explants per treatment). Rooting response was evaluated based on the number of roots per shoot (RN) and root length (RL). The number of roots was determined by counting the roots produced by each regenerated shoot, and root length was measured in centimeters (cm).

#### 4.3.3. Acclimatization of Regenerated Plants

Following root development, regenerated bulblets/plantlets were removed from the culture vessels and transferred to an acclimatization substrate consisting of peat moss and perlite at a 1:1 ratio. The plants were maintained under acclimatization conditions, and after approximately one month, healthy and successfully acclimatized plants were evaluated.

### 4.4. In Vitro Culture Conditions

MS medium was used as the basal medium for all establishment and regeneration in vitro cultures. All prepared media were solidified with 0.65% (*w*/*v*) agar and contained 3% (*w*/*v*) sucrose. Media pH was adjusted to 5.7–5.8 with 1 N NaOH or 1 N HCl before autoclaving (121 °C, 20 min). MS medium, PGRs, AgNO_3_, and chemicals were sourced from Duchefa. 5% commercial NaOCl was used for the sterilization stage, and 0.05% Tween 20 was used as the surfactant. Cultures were incubated at 21 ± 2 °C under a 16 h light/8 h dark photoperiod at ~40 μmol m^−2^ s^−1^ provided by cool-white fluorescent lamps.

### 4.5. Experimental Design and Statistical Analysis

Experiments 1–4 were conducted using factorial treatment arrangements in a CRD, with three independent replicates and five explants per replicate. Thus, each treatment included 15 cultured explants. Experiment 5 consisted of 18 treatment combinations representing two cytokinin types (BAP and TDZ), three cytokinin concentrations, and three NAA concentrations, with three independent replicates and three explants per replicate, corresponding to nine explants per treatment. Experiment 6 consisted of six treatment combinations involving two basal medium strengths and three NAA concentrations, with three independent replicates and three explants per replicate. Observations for experiments 1–4 were recorded 7 days after cultivation, and for experiments 5 & 6, they were recorded 8 weeks after cultivation. The recorded variables included contamination rate during explant establishment, number of regenerated bulblets per explant, shoot length, regeneration percentage, number of roots, and root length. All statistical analyses were performed using SPSS version 27.0. For Experiments 5 and 6, three independent replicate vessels were used for each treatment, with three explants per vessel. For each replicate vessel, the maximum response observed among the three explants was recorded as the replicate-level observation. Treatment means were calculated from the three replicate-level observations. Duncan’s multiple range test (DMRT) was used for multiple comparisons at the 1% significance level (*p* ≤ 0.01).

## 5. Conclusions

The present study established a species-specific in vitro regeneration protocol for the native Iranian tulip, *Tulipa montana* var. *chrysantha*. Explant establishment was optimized by evaluating explant type, sodium hypochlorite exposure time, and AgNO_3_ concentration. Among the AgNO_3_ treatments tested during establishment, 300 mg L^−1^ resulted in the lowest contamination and was subsequently retained during regeneration; therefore, its independent effect on regeneration cannot be determined. Bulb-scale explants were responsive to regeneration, whereas leaf, petal, and flowering-stem explants did not regenerate under the tested conditions. Exogenous cytokinin–auxin combinations affected bulblet regeneration, with the highest observed number of regenerated bulblets (3.66 ± 0.57 per explant) obtained with 2 mg L^−1^ TDZ + 1 mg L^−1^ NAA. For rooting, ½ MS + 1 mg L^−1^ NAA produced the highest number of roots per shoot (5.00 ± 0.00), whereas ½ MS + 0.5 mg L^−1^ NAA produced the longest roots (0.46 ± 0.05 cm). Approximately 80% of regenerated plantlets survived after one month of acclimatization. Because histological analyses were not performed, the regeneration pathway cannot be conclusively classified as direct organogenesis. Overall, the protocol provides a basis for in vitro regeneration and conservation-oriented propagation of *T. montana* var. *chrysantha*. Further studies addressing genetic fidelity, regeneration pathway, rooting percentage, and long-term ex vitro performance would strengthen the application of this protocol for conservation and horticultural purposes.

## Figures and Tables

**Figure 1:**
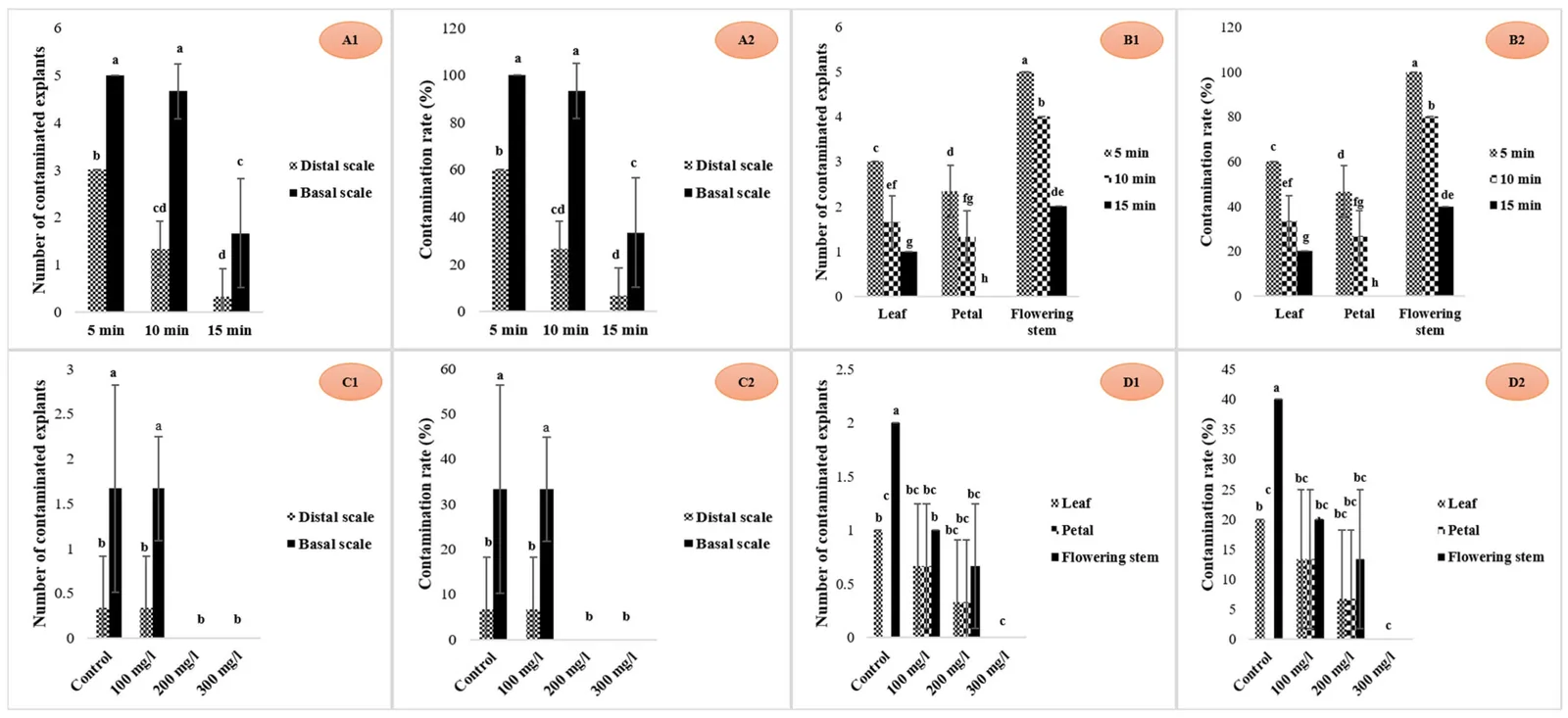
Effect of sodium hypochlorite exposure time and silver nitrate concentration on contamination of *Tulipa montana* var. *chrysantha* explants during in vitro establishment. (**A1**,**A2**) Exp 1: Effect of 2.5% sodium hypochlorite exposure time (5, 10, and 15 min) on contamination of distal and basal bulb-scale explants, (**A1**) number of contaminated explants, (**A2**) contamination rate (%); (**B1**,**B2**) Exp 2: effect of 1.5% sodium hypochlorite exposure time on contamination of leaf, petal, and flowering-stem explants, (**B1**) number of contaminated explants, (**B2**) contamination rate (%); (**C1**,**C2**) Exp 3: effect of silver nitrate (AgNO_3_; 0, 100, 200, and 300 mg L^−1^) on contamination of distal and basal bulb-scale explants, (**C1**) number of contaminated explants, (**C2**) contamination rate (%); (**D1**,**D2**) Exp 4: effect of AgNO_3_ concentration on contamination of leaf, petal, and flowering-stem explants, (**D1**) Number of contaminated explants, (**D2**) contamination rate (%). Data are presented as means with error bars (mean ± standard deviation (M ± STD)). Different lowercase letters indicate significant differences among treatments, as determined by Duncan’s multiple range test (DMRT) at *p* ≤ 0.05.

**Figure 2:**
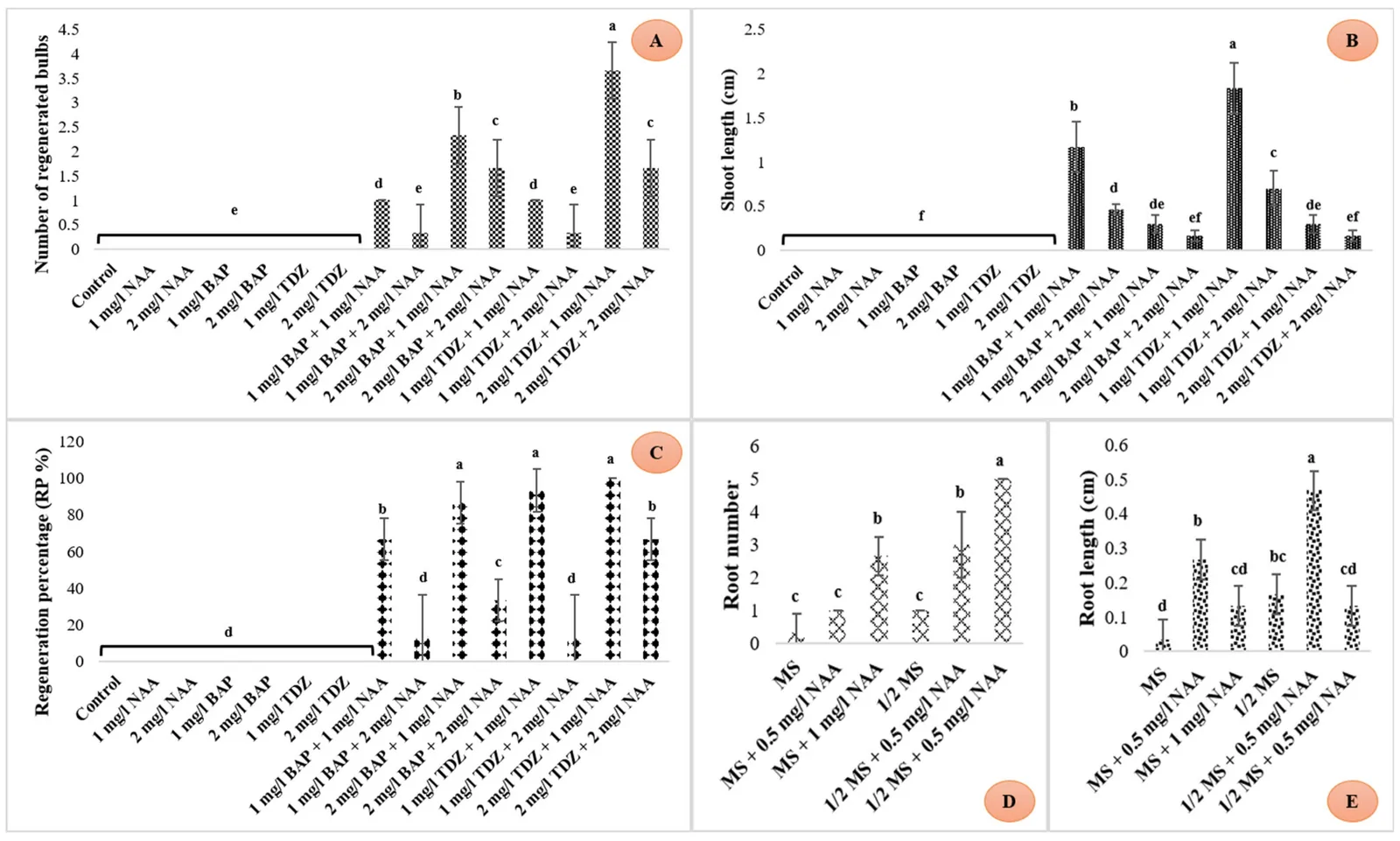
Effects of plant growth regulator treatments on in vitro regeneration and rooting of *Tulipa montana* var. *chrysantha* basal bulb scales. (**A**) Number of regenerated bulblets (NB), (**B**) shoot length (SL, cm), and (**C**) regeneration percentage (RP%) under different concentrations of BAP or TDZ in combination with NAA. (**D**) Root number (RN) and (**E**) root length (RL, cm) of regenerated bulblets cultured on MS or ½ MS medium supplemented with different concentrations of NAA. Data are presented as means with error bars (mean ± standard deviation (M ± STD)). Different lowercase letters indicate significant differences among treatments compared using Duncan’s multiple range test (DMRT) at *p* ≤ 0.05.

**Figure 3:**
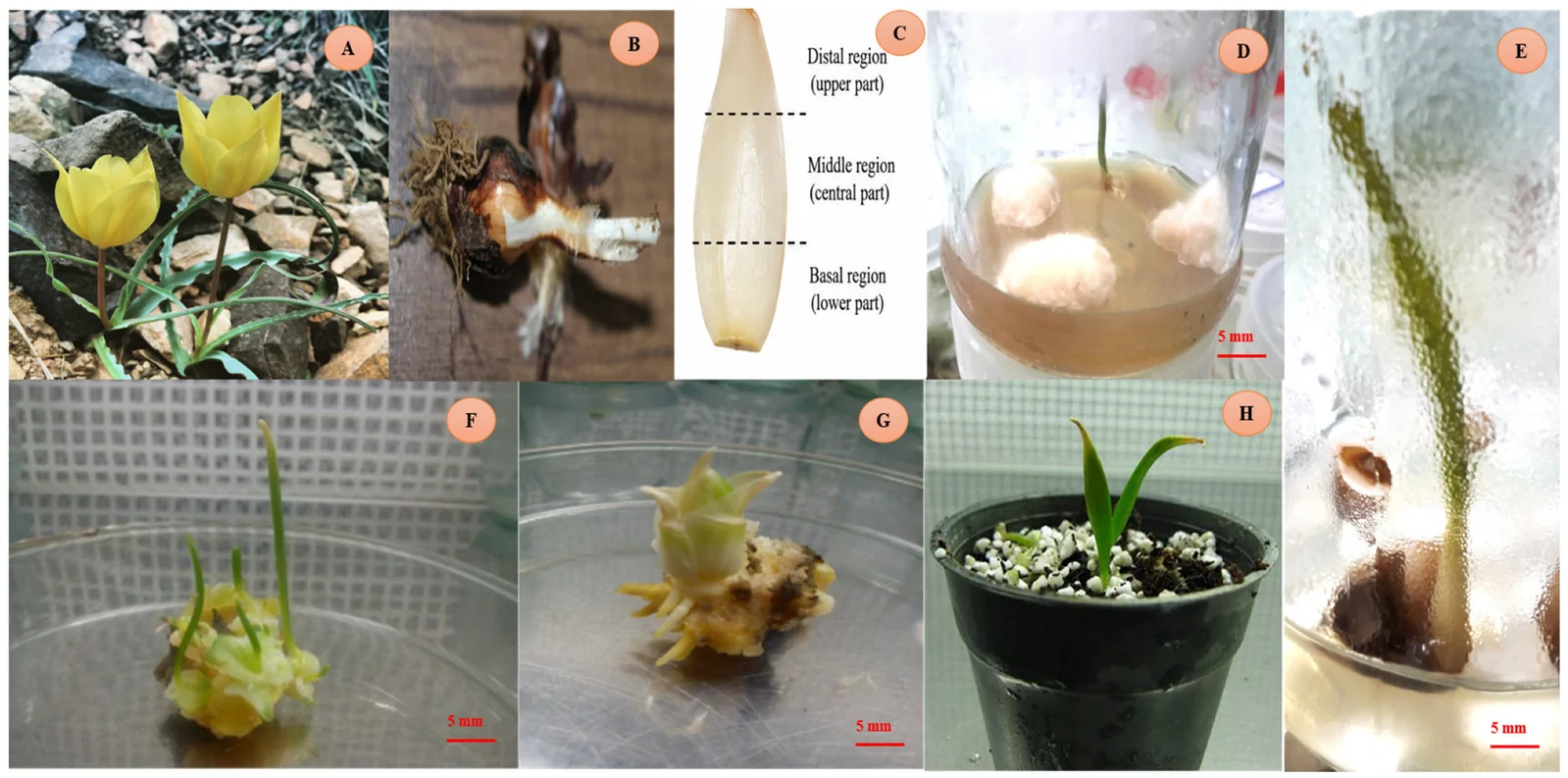
In vitro regeneration and acclimatization of *Tulipa montana* var. *chrysantha*. (**A**) Natural habitat of *T. montana* in the Alborz Mountains of Iran; (**B**) bulbs collected from their natural habitat; (**C**) different regions of tulip bulbs which were used as explants during the experiments; (**D**) initial establishment and contamination of explants under in vitro conditions; (**E**) development of in vitro shoots from newly regenerated bulbs; (**F**) shoot and bulblet regeneration from basal bulb-scale explants; (**G**) regenerated plantlet with developing roots; and (**H**) acclimatized regenerated plant growing under ex vitro conditions.

## Data Availability

The original contributions presented in the study are included in the article; further inquiries can be directed to the corresponding author.

## Abbreviations

The following abbreviations are used in this manuscript:

BAP	6-Benzylaminopurine
CRD	Completely Randomized Design
MS	Murashige & Skoog
NAA	1-Naphthaleneacetic acid
NB	Newly formed bulbs
TDZ	Thiadiazuron
PGRs	Plant Growth Regulators
RP	Regeneration Percentage
RL	Root Length
RN	Root Number
SL	Shoot Length
